# Screening, purification and characterization of cellulase from cellulase producing bacteria in molasses

**DOI:** 10.1186/s13104-018-3558-4

**Published:** 2018-07-04

**Authors:** Farjana Islam, Narayan Roy

**Affiliations:** 1 Department of Biochemistry and Molecular Biology, University of Rajshahi, Rajshahi, 6205 Bangladesh; 2 Department of Biochemistry and Molecular Biology, Bangabandu Sheikh Mujibur Rahman Science and Technology University, Gopalganj, 8100 Bangladesh

**Keywords:** Cellulase, Cellulose, Molasses, Bacteria, Screening, Purification and characterization

## Abstract

**Objectives:**

This study was conducted to isolate, screening and purification of cellulase from bacteria present in sugar industry waste (molasses) and characterization by morphological and biochemical analysis.

**Results:**

Based on experiments, three bacterial strains produced clear transparent zone into carboxymethyl cellulose (CMC) agar plate were identified as cellulase producing bacteria. Different culture parameters such as pH, temperature, incubation period, substrate concentration and carbon sources were optimized for enzyme production. According to the morphological and biochemical tests, the isolated strains were identified as *Paenibacillus* sp., *Bacillus* sp. and *Aeromonas* sp. The first strain *Paenibacillus* sp. showed high potentiality for maximum cellulase production (0.9 µmol ml^−1^ min^−1^) at pH 7.0 after 24 h of incubation at 40 °C in a medium containing 1.0% CMC. Then *Paenibacillus* sp. was selected for enzyme purification by ammonium sulfate precipitation, DEAE-cellulose and CM-cellulose column chromatography, respectively. In last step of purification, specific activity, recovery and purification fold were 2655 U/mg, 35.7% and 9.7, respectively. The molecular weight of the purified cellulase was found to be 67 kDa by SDS-PAGE, had an optimal pH and temperature at 7.0 and 40 °C. According to substrate specificity, the purified cellulase had high specificity on CMC substrate which indicated it to be an endo-β-1,4-glucanase.

**Electronic supplementary material:**

The online version of this article (10.1186/s13104-018-3558-4) contains supplementary material, which is available to authorized users.

## Introduction

Cellulose is a homopolymer of d-glucose units linked by β-1,4 bonds [1]. It is the most abundant biomass and a major structural component of plants and a renewable source of energy in the biosphere [2–4]. Therefore, it has become considerable economic interest to develop suitable processes for effective treatment and utilization of cellulose containing wastes as cheap carbon sources [5]. Cellulose is mainly degraded by cellulase enzyme which is commonly produced by bacteria and fungi [6]. The cellulases can effectively hydrolyze cellulose into glucose unit via the synergistic actions of the enzymes, known as endo-β-1,4 glucanase, cellobiohydrolase and β-D-glucosidase [7].

Cellulases have attracted much interest because of their diverse application in textile, detergent, leather, food, feed and paper industries [4, 8–11]. It is also used in biomass fermentation, fiber modification and in pharmaceutical purposes [5, 12]. Application of these enzymes in such industries demands the identification of stable enzymes that can active at high pH and increased temperature [9]. The growth rate of bacteria is faster than fungi and has been widely used in cellulase production under different culture conditions [13].

For many years, several studies on isolation and characterization of cellulose degrading bacteria from industrial wastes indicated that only a small number of bacteria can produces large amount of bioactive compounds that are capable of complete hydrolysis of crystalline cellulose in vitro [2, 14]. Among different industrial wastes, sugar industry wastes such molasses are mainly cellulosic in nature and the microorganisms present there have the capacity to degrade cellulose into glucose units for their normal growth and development [15]. Therefore, this study was conducted to screening, optimization, purification and characterization of cellulase from cellulase producing bacteria present in molasses.

## Main text

### Materials and methods

#### Isolation and screening of cellulase producing bacteria

Molasses were collected from a sugar industry area located in Katakhali region of Rajshahi city area in Bangladesh. The collected sample (1 g) was led to serial dilution. The diluted sample up to 10^−6^ was taken into Luria-Bertani (LB) medium. Then 100 µl of the solution was transferred into 1l of carboxymethyl cellulose (CMC) agar media plates containing 0.5 g KH_2_PO_4_, 0.25 g MgSO_4_, 0.25 g cellulose and 2 g gelatin for the enhancement of the bacterial activity. The plates were then incubated at 37 °C for overnight and preserved at 4 °C [16]. The enzyme activity was confirmed by different types of methods such as congo red, iodine solution and filter paper degradation method [17, 18]. The bacterial isolates were inoculated in a basal salt medium containing filter paper for their cellulytic activity test.

### Identification of cellulytic bacteria by morphological and biochemical characterization

#### Morphological characterization

The plates were examined for Gram staining and microscopic viewing for identification of bacterial strains [19]. This technique was used to distinguish the gram positive and gram negative bacteria.

#### Biochemical characterization

The bacterial isolates were identified by performing several biochemical tests like Fermentation test, Catalase test, Citrate utilization test, Methyl-red test, H_2_S production and Voges–Proskauer test by standard methods [20].

### Optimization of culture conditions on cellulase activity

To determine the effects of pH, temperature, incubation period, substrate concentration and carbon sources on cellulase production, selected bacterial isolates were grown in CMC broth media and tested at various parameters. The effects of all factors on enzyme activity were determined by measuring the cellulase activity at different pH values (5–11) and the temperature (20–45 °C) and the incubation period (24–96 h) at 37 °C. The various concentrations of CMC substrate (0.5–2%) were used to get the maximum cellulase production. Carbon sources have been replaced by various substances. Applied carbon sources were xylose, oat spelt xylan, rich bran xylan, starch, carboxymethyl cellulose, cellobiose, wheat bran xylan and chitin. Different types of nitrogen sources metal salts were used to observe the effects on growth and enzyme production (data not shown).

### Enzyme assay

#### Preparation of crude enzyme

The isolate that showed a maximum zone of hydrolysis was cultured in LB broth medium and incubated at 37 °C for overnight. Then the cultures were centrifuged and clear supernatant was used as a source of crude enzyme solution.

### Endo-β-1,4-glucanase activity assay by DNS method

Endo-β-1,4-glucanase activity of cellulase was measured by DNS (3,5-dinitrosalicylic acid) method through the amount of reducing sugars liberated during hydrolysis [21]. 1% solution of CMC was prepared in 1 N citrate buffer (pH 5.0) and was considered as substrate. 100 µl crude enzymes and 1 ml citrate buffer were added into the mixture of 1 ml CMC solution. The mixture was incubated at 45 °C for 30 min. Then DNS was added to the solution to stop the reaction [21]. The treated samples were boiled for 10 min, cooled in water for color stabilization and the optical density was measured at 540 nm. One unit of endo-β-1,4-glucanase activity was defined as the amount of enzyme that could hydrolyze CMC and release 1 µmol of glucose within 1 min of reaction [5].

### Purification of cellulase

#### Ammonium sulfate precipitation

The prepared crude enzyme was brought to 80% saturation with solid ammonium sulphate. The mixture was kept overnight at 4 °C in a magnetic stirrer. Then the mixture was centrifuged and the pellet was dissolved in 50 mM sodium phosphate buffer saline at pH 7.0 for further purification. The partially purified enzyme was dialyzed against the phosphate buffer.

#### DEAE-cellulose column chromatography

60 ml of the enzyme sample was applied to DEAE-cellulose (Diethylaminoethyl cellulose) column which was equilibrated with 10 mM Tris-Hcl buffer at pH 7.0. The collected unbound eluted fraction was used for measuring the enzyme activity at 540 nm and protein concentration at 280 nm. The fraction showing high activity was pooled and kept for SDS-PAGE (sodium dodecyl sulfate polyacrylamide gel electrophoresis) analysis.

#### CM-cellulose column chromatography

55 ml of DEAE unbound solution was applied to CM-cellulose column which was equilibrated with 10 mM Tris-HCl buffer at pH 7.0. Proteins were eluted by gradually increasing of NaCl gradient from 0.0 to 0.3 M with the same buffer. After the enzyme activity assay, the eluted fraction was stored for SDS-PAGE analysis.

### Protein estimation and molecular weight determination

Protein concentrations in the crude sample were estimated by using Lowry method with bovine serum albumin (BSA) as a standard [22] and SDS-PAGE was used for molecular weight determination [23]. The standard proteins markers were loaded next to the purified protein, followed by the crude and dialyzed sample.

### Determination of substrate specificity

The various polysaccharides like carboxymethyl cellulose, xylose, oat spelt xylan, rich bran xylan, cellulose, lactose and wheat bran xylan was used to determine the substrate specificity of the purified cellulase from strain C_1_ [16].

## Results

### Isolation, screening and identification of cellulase producing bacteria

The biochemical characterizations of the isolated strains (C_1_, C_2_ and C_3_) are presented in Additional file 3: Supplementary Table 1. A microscopic examination revealed that the isolated strain C_1_ and C_2_ was rod shaped and found to be gram positive where as C_3_ strain was short rod in shape and found to be gram negative. Based on both biochemical and morphological characteristics, the isolated strains were identified to be *Paenibacillus* sp. (C_1_), *Bacillus* sp. (C_2_) and *Aeromonas* sp. (C_3_), respectively, where C_1_ and C_2_ was gram positive and C_3_ was gram negative bacteria. In filter paper degradation assay, all strains were found to completely degrade the filter paper with incubation period of 7 days (Additional file 1: Supplementary Fig. 1), indicates that the isolated strains were cellulytic bacteria.

### Optimization of culture conditions and enzyme activity

**Fig. 1:**
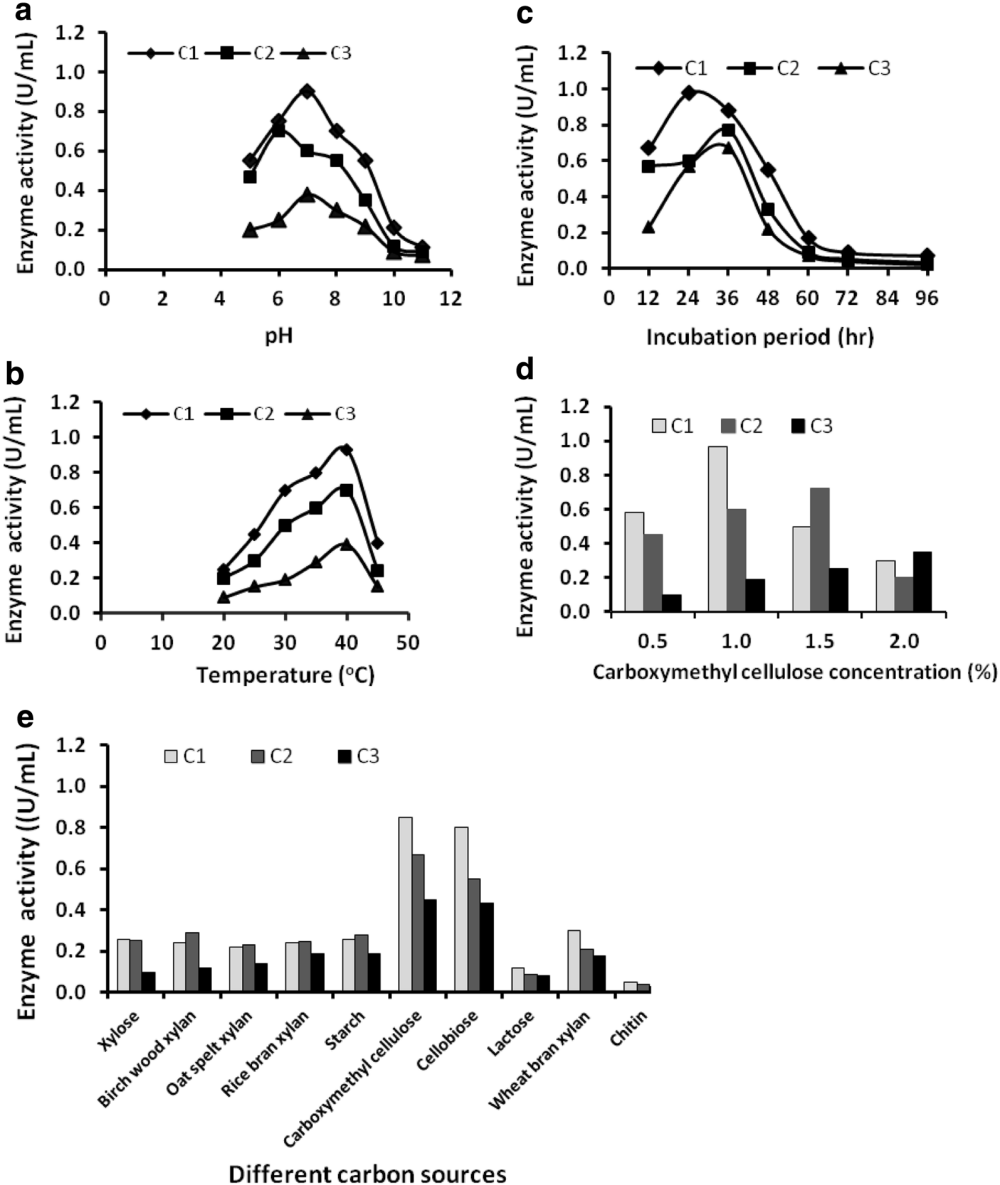
Optimization of cultural parameters and enzyme activity: **a** effect of pH, **b** effect of temperature, **c** effect of incubation period, **d** effect of substrate concentration and **e** effect of carbon sources on cellulase production

The optimization of culture conditions on cellulase production are presented in Fig. 1. At neutral pH, the C_1_ strain showed highest enzyme activity (0.90 µmol ml^−1^ min^−1^) at 40 °C. The C_1_ isolate produced maximum amount of cellulase and showed highest enzyme activity (0.98 and 0.97 µmol ml^−1^ min^−1^) on 24 h incubation period at 1% CMC concentration, respectively. Among different carbon sources, CMC was found to be the most suitable one for the organism growth as well as better cellulase production. Peptone as a nitrogen sources and CaCl_2_ as a metal salt showed the profound effect on cellulase production (data not shown).

### Purification of cellulase

Details for the cellulase purification are presented in Table 1. The crude enzyme extract contained 300 mg protein showed 81,788 U/l in terms of total activity. In the final stage of purification with CMC column chromatography, the specific activity, yield and purification fold were 2655 U/mg, 35.7% and 9.7, respectively.

**Table 1:** Purification of cellulase from crude sample of *Paenibacillus* sp.

Purification steps	Volume (ml)	Total protein (mg)	Total activity (U)	Specific activity (U/mg)	Yield (%)	Purification fold
Crude extract	1500	300	81,788	273	100	1
80% ammonium sulfate saturation	60	160	75,121	469	91.8	1.7
DEAE-cellulose column chromatography	55	40	64,525	1613	78.9	5.9
CM-cellulose column chromatography	25	17	29,210	1718	35.7	9.7

### Molecular weight determination

**Fig. 2:**
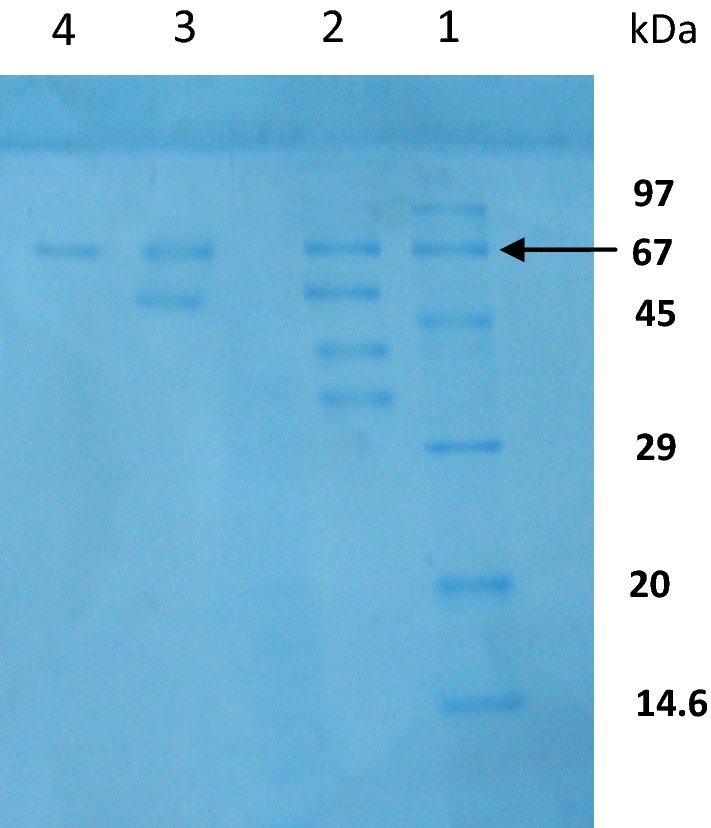
Molecular weight determinations by SDS-PAGE method. Lane 1, marker protein: Phosphorylase B (97 kDa), Bovine serum albumin (67 kDa), Ovalbumin (45 kDa), Carbonic anhydrase (29 kDa), Trypsin inhibitor (20 kDa) and Lysozyme (14.6 kDa). Lane 2, crude enzyme. Lane 3, DEAE-cellulose unbound fraction and Lane 4, CM-cellulose bound (purified protein). The migration position of cellulase is indicated as 67 kDa

The molecular weight of the enzyme was found to be 67 kDa comparing with the marker proteins (Fig. 2).

### Substrate specificity

The purified cellulase from strain C_1_ showed the highest activity against CMC and low activity to wheat bran xylan, lactose, rice xylan, xylose and oat spelt xylan (Additional file 2: Supplementary Fig. 2). According to substrate specificity, the purified cellulase had high specificity on CMC which indicated it to be an endo-β-1,4-glucanase.

## Discussion

The cellulytic activities of the isolated bacterial strains depend on the sources and amount of biowaste in natural environments [24]. In present study, the isolated strains were characterized by morphological and biochemical analysis [19] and then screened for their cellulase activity by Congo red iodine solution and DNS test [21, 25]. The isolated strains were identified as *Paenibacillus* sp., *Bacillus* sp. and *Aeromonas* sp., respectively. The strains were inoculated in fermentation medium and cellulase production was assayed [19, 26]. Enzyme production by the selected strains was tested with different culture conditions [27, 28].

In present study, *Paenibacillus* sp. had maximum cellulase activity at pH 7.0 and 40 °C temperature on 24 h incubation period at 1% CMC substrate concentration (Fig. 1 and Additional file 2: Supplementary Fig. 2). In a previous study, the purified cellulase from *Paenibacillus* sp. E2 and E4 in paper mill sludges showed high enzymatic activity on CMC substrate and filter paper degradation [29].

To characterize the purified enzyme specificity, we initially use qualitative tests like CMC test and filter paper degradation test. CMC agar allows us to identify isolates with cellulase activity on soluble cellulose such as CMC thus representing mainly endoglucanase and beta- glucosidase activities. Secondly, we then screened isolates displaying cellulase activity on CMC for activity on crystalline insoluble cellulose such as filter paper (Additional file 1: Supplementary Fig. 1). In other tests, the purified enzyme showed high activity to CMC and lower activity on xylose, rice xylan and wheat bran xylan (Additional file 2: Supplementary Fig. 2). The high activity on CMC suggests that the CM Case purified from *Paenibacillus* sp. might be an endoglucanase on the basis of definition by Coughlan and Mayer [30]. Thus, in present study, substrate specificity indicates that the purified enzyme might be an endo-β-1,4-glucanase. Researchers have also characterized a novel endoglucanase (Cel9P) based on CMC substrate specificity from a newly isolated *Paenibacillus* sp. BME-14 [31] and a novel thermophilic, cellulose-degrading bacterium *Paenibacillus* sp. strain B39 from poultry manure compost [32].

Considering highest cellulase activity, *Paenibacillus* sp. strain was selected for enzyme purification, molecular weight determination and enzyme characterization [33]. In the last stage of enzyme purification, the specific activity, yield and purification fold were 2655 U/mg, 35.7% and 9.7, respectively (Table 1). The molecular weight of the purified protein was 67 kDa (Fig. 2) and the enzyme was characterized at pH 7.0 and 40 °C temperature against different substrates. Among different substrates, CMC was found as a prominent source for maximum endo-glucanase production and at optimized conditions the purified enzyme was stable and didn’t lose the activity. In present study, all the cellulase positive isolated strain may be an integral part of our future work to develop an efficient cellulase producing systems which can be used for industrial purposes.

## Conclusion

Results of this study indicate that cellulase producing bacterial strains can be grown at different optimized conditions. The isolated strain *Paenibacillus* sp. showed maximum cellulase activity at pH 7.0 and 40 °C temperature on 24 h incubation period at 1% CMC substrate concentration. According to substrate specificity, the purified cellulase showed high specificity on CMC which indicated it to be an endo-β-1,4-glucanase. The purified cellulase might be useful for several industrial applications.

### Limitation

There are two limitations of present study. First, the molasses sample has been collected only from a single sugar industry area and secondly the molecular characterization of the purified cellulose was not performed because of limited lab facilities.

## Additional files

**Additional file 1.** Supplementary Figure 1: Filter paper degradation by C (control), C_1_, *(Paenibacillus sp*.), C_2_ (*Bacillus sp*.) and C_3_ (*Aeromonas sp.)* strains respectively.

**Additional file 2.** Supplementary Figure 2: Characterization of cellulase from *Paenibacillus sp.*: Effect of pH (A), Effect of temperature (B) and Effect of different substrate (C) on enzyme activity.

**Additional file 3.** Supplementary Table 1: Physiological and biochemical characteristics of the isolated bacterial strains.

## Abbreviations

CMC: carboxymethyl cellulose
LB: Luria–Bertani
DNS: 3,5-dinitrosalicylic acid

## References

[1] Romeo T (2008). Bacterial biofilms.

[2] Saha S, Roy R, Sen SK, Ray AK (2006). Characterization of cellulase-producing bacteria from the digestive tract of tilapia, *Oreochromis mossambica* (Peters) and grass carp, *Ctenopharyngodon idella* (Valenciennes). Aquac Res.

[3] Klemm D, Heublein B, Fink H-P, Bohn A (2005). Cellulose: fascinating biopolymer and sustainable raw material. Angew Chem Int Ed.

[4] Bhat MK (2000). Cellulases and related enzymes in biotechnology. Adv Biotechnol.

[5] Shanmugapriya K, Saravana PS, Krishnapriya MM, Mythili A, Joseph S (2012). Isolation, screening and partial purification of cellulose from cellulose producing bacteria. Int J Adv Biotechnol Res.

[6] Immanuel G, Dhanusha R, Prema P, Palavesam A (2006). Effect of different growth parameters on endoglucanase enzyme activity by bacteria isolated from coir retting effluents of estuarine environment. Int J Environ Sci Technol.

[7] Perez J, Munoz-Dorado J, de la Rubia T, Martinez J (2002). Biodegradation and biological treatments of cellulose, hemicellulose and lignin: an overview. Int Microbiol.

[8] Gyalai-Korpos M, Nagy G, Mareczky Z, Schuster A, Réczey K, Schmoll M (2010). Relevance of the light signaling machinery for cellulase expression in *Trichoderma reesei* (Hypocrea jecorina). BMC Res Notes.

[9] Abdelnasser SSI, Ahmed IE (2007). Isolation and identification of new cellulases producing thermophilic bacteria from an Egyptian hot spring and some properties of the crude enzyme. Aust J Basic Appl Sci.

[10] Chandara SKR, Snishamol C, Prabhu NG (2005). Cellulase production by native bacteria using water hyacinth as substrate under solid state fermentation: Malaysian. J Microbiol.

[11] Cavaco-Paulo A (1998). Mechanism of cellulose action in textile processes. Carbohydr Polym.

[12] Cherry JR, Fidantsef AL (2003). Directed evolution of industrial enzymes: an update. Curr Opin Biotechnol.

[13] Nakamura K, Kappamura K (1982). Isolation and identification of crystalline cellulose hydrolyzing bacterium and its enzymatic properties. J Ferment Technol.

[14] Doi RH (2008). Cellulase of mesophilic microbes: cellulosome and non-cellulosome producers. Ann NY Acad Sci.

[15] Rasul F, Afroz A, Rashid U, Mehmood S, Sughra K, Zeeshan N (2015). Screening and characterization of cellulase producing bacteria from soil and waste (molasses) of sugar industry. Int J Biosci.

[16] Yin LJ, Huang PS, Lin HH (2010). Isolation of cellulase producing bacteria and characterization of the cellulase from the isolated bacterium *Cellulomonas* sp. YJ5. J Agric Food Chem.

[17] Ando T, Chambost JP, Kotoujansky A, Cattano J, Barras F (1984). Mutants of Erwiniachrysanthemi defective in secretion of pectinase and cellulase. J Bacteriol.

[18] Hong JH, Kim JY, Hur SH (2005). Purification and characterization of an alkaline cellulase from a newly isolated alkalophilic *Bacillus* sp. HSH-810. Biotech Lett.

[19] Apun K, Jong BC, Salleh MA (2000). Screening and isolation of a cellulolytic and amylolytic *Bacillus* sp. from pith waste. J Gen Appl Microbes.

[20] Buchanan RE, Gibbons NE (1974). Bergey’s of determinative bacteriology.

[21] Miller GL (1959). Use of Dinitrosalisylic acid reagent for determination of reducing sugars. Anal Chem.

[22] Lowry OH, Rosebrough NJ, Farr AL, Randall RJ (1951). Protein measurement with the Folinciocalteu’s reagent. J Biol Chem.

[23] Haung XP, Monk C (2004). Purification and characterization of a cellulase from a newly isolated thermophilic aerobic bacterium *Caldibacillus cellulovorans* gen. nov. sp.. World J Microbiol Biotechnol.

[24] Gopinath SM, Shareef I, Ashalatha Ranjit S (2012). Isolation, screening and purification of cellulase from cellulase producing *Klebsiella variicola* RBER3 (KF036184.1). Int J Sci Res.

[25] Irfan M, Safdar A, Syed Q, Nadeem M (2012). Isolation and screening of cellulolytic bacteria from soil and optimization of cellulase production and activity. Turk J Biochem.

[26] Chundakkadu K (1998). Production of bacterial cellulases by solid state fermentation of banana wastes. Bioresour Technol.

[27] Nandimath AP, Kharat KR, Gupta SG, Kharat AS (2016). Optimization of cellulase production for *Bacillus* sp. and *Pseudomonas* sp. soil isolates. Afr J Microbiol Res.

[28] Lynd LR, Weimer PJ, van Zyl WH, Pretorius IS (2002). Microbial cellulose utilization: fundamentals & biotechnology. Microbiol Mol Biol Rev.

[29] Maki ML, Broere M, Leung KT, Qin W (2011). Characterization of some efficient cellulase producing bacteria isolated from paper mill sludges and organic fertilizers. Int J Biochem Mol Biol.

[30] Coughlan MP, Mayer F, Balows A, Truper HG, Dworkin M, Harder W, Schleifer KH (1992). The cellulose-decomposing bacteria and their enzyme systems. The prokaryotes.

[31] Fu X, Liu P, Lin L, Hong Y, Huang X, Meng X, Liu Z (2010). A novel endoglucanase (Cel9P) from a marine bacterium *Paenibacillus* sp. BME-14. Appl Biochem Biotechnol.

[32] Wang CM, Shyu CL, Ho SP, Chiou SH (2008). Characterization of a novel thermophilic, cellulose-degrading bacterium *Paenibacillus* sp. strain B39. Lett Appl Microbiol.

[33] Lee YJ, Kim BK, Lee BH, Jo KI, Lee NK (2006). Purification and characterization of cellulase produced by *Bacillus amyloliquefaciens* DL-3 utilizing rice hull. Coll Nat Resour Life Sci.

