# Supplementary figures and images for: Screening, purification and characterization of cellulase from cellulase producing bacteria in molasses
Source: BMC Res Notes. 2018 Jul 4;11:445. doi: 10.1186/s13104-018-3558-4 (PMC6032522; doi:10.1186/s13104-018-3558-4)

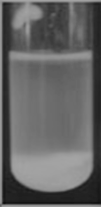

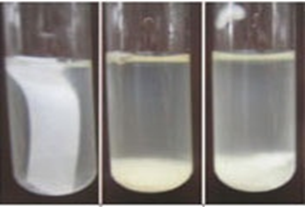


C

C11

C2

C3

**Supplementary Figure 1**

Supplement: Supplementary file 1 — Additional file 1. Supplementary Figure 1: Filter paper degradation by C (control), C1, (Paenibacillus sp.), C2 (Bacillus sp.) and C3 (Aeromonas sp.) strains respectively. [file 13104_2018_3558_MOESM1_ESM.doc]
