# Supplementary material for: Screening, purification and characterization of cellulase from cellulase producing bacteria in molasses
Source: BMC Res Notes. 2018 Jul 4;11:445. doi: 10.1186/s13104-018-3558-4 (PMC6032522; doi:10.1186/s13104-018-3558-4)
Supplement: Supplementary file 2 — Additional file 2. Supplementary Figure 2: Characterization of cellulase from Paenibacillus sp.: Effect of pH (A), Effect of temperature (B) and Effect of different substrate (C) on enzyme activity. [file 13104_2018_3558_MOESM2_ESM.doc]

**C**

**B**

**A**

**A**

**C**

**Supplementary Figure 2**
