# Supplementary material for: Screening, purification and characterization of cellulase from cellulase producing bacteria in molasses
Source: BMC Res Notes. 2018 Jul 4;11:445. doi: 10.1186/s13104-018-3558-4 (PMC6032522; doi:10.1186/s13104-018-3558-4)
Supplement: Supplementary file 3 — Additional file 3. Supplementary Table 1: Physiological and biochemical characteristics of the isolated bacterial strains. [file 13104_2018_3558_MOESM3_ESM.doc]

**Supplementary Table 1** Physiological and biochemical characteristics of the isolated bacterial strains

| Characteristics | C_1_ | C_2_ | C_3_ |
| --- | --- | --- | --- |
| Gram staining | + | + | - |
| Morphology | Rod | Rod | Short rod |
| Motility test | + | + | + |
| Colony color (visual test) | White | White | White |
| Glucose Fermentation | + | + | + |
| Galactose Fermentation | + | + | + |
| Sucrose Fermentation | + | + | + |
| Catalase test | + | + | + |
| Citrate Utilization | - | + | + |
| Methyl-Red test | + | - | + |
| H_2_S Production test | - | - | - |
| Voges-Proskauer test | - | + | - |
